# Editorial: Cross-Frontier Communication: Phytohormone Functions at the Plant-Microbe Interface and Beyond

**DOI:** 10.3389/fpls.2020.00386

**Published:** 2020-04-08

**Authors:** Susanne Berger, Saskia C. M. Van Wees, Ole Nybroe, Dominik K. Großkinsky

**Affiliations:** ^1^Julius-Von-Sachs-Institute of Biosciences, Biocenter, Pharmaceutical Biology, University of Würzburg, Würzburg, Germany; ^2^Plant-Microbe Interactions, Department of Biology, Faculty of Science, Utrecht University, Utrecht, Netherlands; ^3^Department of Plant and Environmental Sciences, Faculty of Science, University of Copenhagen, Frederiksberg, Denmark; ^4^Center for Health and Bioresources, Bioresources Unit, AIT Austrian Institute of Technology GmbH, Tulln an der Donau, Austria

**Keywords:** bacteria, fungi, inter-kingdom communication, phytohormone, plant-microbe interaction, signaling, virus

Phytohormones are central signaling molecules that are essential for the regulation of plant growth and development as well as for plant responses to biotic and abiotic stresses (Pieterse et al., 2012; Großkinsky et al., 2016). Increasing insights into multifaceted plant-microbe interactions underpin the relevance of phytohormones that are produced or modulated by the plant and/or the plant-associated pathogenic and beneficial microbes. Several phytohormones have been suggested as important components in inter-kingdom communication (Leach et al., 2017), impacting plants as well as microbes. In this Research Topic, we aimed to increase knowledge of phytohormone functions at the plant-microbe interface and their relevance for plants and microbes at the fundamental and applied scientific level. A better understanding of their multiple roles in managing plant defenses and fitness (Vos et al., 2013; Guo et al., 2018; van Butselaar and Van den Ackerveken, in press) will facilitate targeting of the phytohormone network for applied approaches in plant cultivation and protection. This could include engineering of phytohormonal crosstalk (Shigenaga et al., 2017) or development of phytohormone-based microbial biocontrol solutions.

All phytohormones have been covered in three reviews and five original articles related to this Research Topic. Three contributions address several phytohormones and phytohormone interactions, two focus on cytokinins (CKs), two on salicylic acid (SA), and one on brassinosteroids (BRs). The articles cover a broad spectrum of organisms with biotrophic and necrotrophic lifestyles and a broad range of interactions—from beneficial to pathogenic—demonstrating the ubiquitous involvement of phytohormones in plant-microbe interactions.

CKs are classically known for their functions in plant growth and development. More recently, their involvement in a steadily growing number of interactions with microbes has been recognized (Großkinsky and Petrášek, 2019). The review of Akhtar et al. presents an update on the multifaceted roles of CKs in plant-biotic interactions. The (combined) functions of CKs in regulating growth and development, nutritional allocation and plant defense determine the outcome of plant interactions with pathogenic and beneficial bacteria as well as with pathogenic fungi and insect pests. The prospect of CK application in biocontrol is also discussed by the authors. In the paper by Jameson et al. the much-debated function of methylated CKs in infection and virulence of the leaf gall-forming bacterium *Rhodococcus fascians* is investigated. A comprehensive analysis of CKs after infection of pea with virulent and avirulent *R. fascians* strains demonstrated that novel methylated isopentenyladenine-type CKs are uniquely associated with infection by virulent strains. This study provides evidence for specific roles of these microbial CKs in infection establishment and symptom development. Phytoplasmas are bacterial parasites of the phloem tissue, which can cause disease also in economically relevant plants. Dermastia provides an overview on the current knowledge of the involvement of phytohormones in the phytoplasma-plant interaction. Evidence is presented that synthesis of and/or signaling by basically all phytohormones is altered upon infection and that effectors of phytoplasmas can manipulate phytohormone pathways to influence disease development.

Likewise, several oomycetes and fungi produce effectors, which manipulate the pathways of the classical defense hormones SA, jasmonic acid (JA) and ethylene (ET) (Fonseca et al., 2018; Han and Kahmann). However, some effectors can also target phytohormone pathways that primarily function in the regulation of growth and development. Han and Kahmann review the strategies by which effectors of filamentous pathogenic microbes exploit phytohormone pathways for the benefit of the pathogen. This is achieved either by production of hormones/hormone mimics by the pathogen or by proteinaceous effectors that interfere with biosynthesis or signaling in the plant. The authors point out that the SA pathway is the most targeted pathway. One possible strategy to interfere with the SA pathway is by metabolizing this hormone. Along this line, salicylate hydroxylases of the agriculturally relevant pathogen *Fusarium graminearum* were identified in the work of Hao et al.. The SA-metabolizing activity of two enzyme isoforms and their specific relevance for virulence on wheat plants was characterized. Constantin et al. investigated the contribution of SA, JA and ET to resistance of tomato to pathogenic *Fusarium oxysporum* induced by endophytic *F. oxysporum* Fo47. Notably, this endophyte-induced resistance was not associated with priming of hormone-inducible defense gene expression, and mutant analyses demonstrated that it was neither dependent on these three defense phytohormones. Plant defenses against viruses are known to be controlled by SA. Künstler et al. dissected the contribution of the redox regulator glutathione to SA-dependent and -independent defense of tobacco plants against tobacco mosaic virus. They found that glutathione can compensate for SA deficiency to maintain disease resistance. The role of BRs in mycorrhization was investigated by von Sivers et al. using the arbuscular mycorrhizal (AM) fungus *Rhizoglomus irregularis* and transgenic tobacco lines exhibiting altered expression of membrane steroid binding protein 1 (MSBP1), which is suggested to inhibit BR signaling. This work identified BRs able to affect mycorrhization and arbuscular structure, and to directly impact nutrient exchange in the AM symbiosis.

The collection of articles in this Research Topic demonstrates the diversity of functions of phytohormones in various aspects of plant-microbe interactions at multiple levels, which highlights their importance for basic and applied research questions.

## Author Contributions

The authors jointly defined the content of this Research Topic and all participated in the editing process. All authors made a substantial, direct and intellectual contribution to composing this editorial and approved it for publication.

### Conflict of Interest

The authors declare that the research was conducted in the absence of any commercial or financial relationships that could be construed as a potential conflict of interest.

## References

[B1] Fonseca S. Radhakrishnan D. Prasad K. Chini A. (2018). Fungal production and manipulation of plant hormones. Curr. Med. Chem. 25, 253–267. 10.2174/0929867324666170314150827 28292238

[B2] Großkinsky D. K. Petrášek J. (2019). Auxins and cytokinins – the dynamic duo of growth-regulating phytohormones heading for new shores. New Phytol. 221, 1187–1190. 10.1111/nph.15556 30644580

[B3] Großkinsky D. K. van der Graaff E. Roitsch T. (2016). “Regulation of abiotic and biotic stress responses by plant hormones” in Plant Pathogen Resistance Biotechnology, ed D. B. Collinge (Hoboken, NJ: John Wiley & Sons, Inc.), 131–154. 10.1002/9781118867716.ch7

[B4] Guo Q. Major I. T. Howe G. A. (2018). Resolution of growth-defense conflict: mechanistic insights from jasmonate signaling. Curr. Opin. Plant Biol. 44, 72–81. 10.1016/j.pbi.2018.02.009 29555489

[B5] Leach J. E. Triplett L. R. Argueso C. T. Trivedi P. (2017). Communication in the phytobiome. Cell 169, 587–596. 10.1016/j.cell.2017.04.025 28475891

[B6] Pieterse C. M. J. Van der Does D. Zamioudis C. Leon-Reyes A. Van Wees S. C. M. (2012). Hormonal modulation of plant immunity. Annu. Rev. Cell Dev. Biol. 28, 489–521. 10.1146/annurev-cellbio-092910-154055 22559264

[B7] Shigenaga A. M. Berens M. L. Tsuda K. Argueso C. T. (2017). Towards engineering of hormonal crosstalk in plant immunity. Curr. Opin. Plant Biol. 38, 164–172. 10.1016/j.pbi.2017.04.021 28624670

[B8] van Butselaar T. Van den Ackerveken G (in press). Salicylic acid steers the growth-immunity tradeoff. Trends Plant Sci. 10.1016/j.tplants.2020.02.002 32407696

[B9] Vos I. A. Pieterse C. M. J. Van Wees S. C. M. (2013). Costs and benefits of hormone-regulated plant defences. Plant Pathol. 62, 43–55. 10.1111/ppa.12105

